# Elevation of ghrelin by B-adrenergic activation is independent of glucose variations and feeding regimen in the rat

**DOI:** 10.1007/s12020-024-04156-w

**Published:** 2025-04-02

**Authors:** Mayte Alvarez-Crespo, Manuel Gil-Lozano, Yolanda Diz-Chaves, Lucas Carmelo González-Matias, Federico Mallo

**Affiliations:** 1 LabEndoTeam – Laboratory of Endocrinology – Department of Functional Biology and Health Sciences – University of Vigo – Campus as Lagoas – Marcosende, Vigo, Spain; 2 Galicia Sur Health Research Institute (IIS Galicia Sur). SERGAS-UVIGO, Vigo, Spain

**Keywords:** Ghrelin, B-adrenergic, Isoproterenol, Glucose levels, Exendin-4

## Abstract

Ghrelin is a signal involved in the initiation of meals in rodents and humans. Circulating ghrelin levels are elevated before mealwes and reduced after food intake. Several factors have been identified as effective modulators of ghrelin levels. Vagal activation reduced ghrelin in rats, as well as oral carbohydrate and lipid administration in rats and humans. Some hormones, such as incretins, also reduce ghrelin: GLP-1 reduced ghrelin in humans, and Ex4, a GLP-1 receptor agonist, potently inhibited ghrelin in rodents. On the other hand, fasting promotes increases in ghrelin that anticipate the start of meals. We report that beta-adrenergic activation with isoproterenol promotes large acute elevations of circulating ghrelin levels, both in anesthetized and conscious freely-moving rats, either on “ad libitum” feeding or on a fasting regimen.These effects are dose-dependent, caused by intravenous, intraperitoneal, and oral administration, and independent of variations in glucose levels. Pharmacological modulation of β1 and β2 adrenergic receptors with specific agonists and antagonists showed that ghrelin increases are stimulated by β1-adrenergic activation, but also partially by β2-adrenergic activation, suggesting that activation of both is necessary to elicit complete ghrelin elevations. Meanwhile, glucose increases dependent on adrenergic activation appear to be mediated only by β2-adrenergic receptors. In addition, the effects of isoproterenol on increasing ghrelin levels are potent enough to overcome the marked inhibition exerted by exendin-4 that we have previously demonstrated. We also found that administration of isoproterenol in drinking water increases basal ghrelin levels and simultaneous food intake in animals eating ad libitum. Beta-adrenergic activation promotes increases in ghrelin levels in vivo prior to food intake, both in rats eating *ad libitum* and in fasting rats that already have elevated ghrelin levels, in a time- and dose-dependent manner. In addition, the effects of isoproterenol on increasing ghrelin levels are potent enough to overcome the marked inhibition exerted by exendin-4 that we have previously demonstrated. We also found that administration of isoproterenol in drinking water increases basal ghrelin levels and simultaneous food intake in animals eating ad libitum. Beta-adrenergic activation promotes increases in ghrelin levels in vivo prior to food intake, both in eating *ad libitum* and in fasting rats that already have elevated ghrelin levels, in a time- and dose-dependent manner.

## Introduction

Ghrelin has been involved in food intake control since it was isolated and identified as the endogenous ligand for the GHS (Growth-Hormone Secretagogue) receptors (GHSR1). Administration of ghrelin to rodents induces a marked increase in food intake and consequently increased fat accumulation in adipose tissue [1]. Ghrelin is produced and secreted by enteroendocrine cells of the oxyntic glands of the gastric fundus [2, 3], and its levels increase just before meals and during fasting in rodents [1] and humans [4, 5]. In contrast, ghrelin decreased after food intake [2, 4, 5]. Therefore, ghrelin variations are related to the initiation of meals, when it is increased, and to the completion of ingestion, when it is reduced by food intake. In fact, variations in circulating ghrelin levels have also been implicated in alterations in food intake in humans. In general, ghrelin levels appeared to be elevated in negative energy balance states as exercise [6], diabetes mellitus [7], pregnancy [8], starvation [9], anorexia [10] or cachexia [11], and reduced in those states associated with a positive energy balance as general obesity [12] or refeeding. Prader-Willie syndrome would be an exception, as it is a form of obesity in which increased ghrelin levels could be a pathogenic factor contributing to the hyperphagia so characteristic of this disease and related to increased adiposity [12].

Despite the large number of studies in the field, the regulation of ghrelin is not fully understood. Ghrelin levels appear to be reduced after oral administration of carbohydrates, lipids, and proteins in rodents [13] and humans [14]. Lipids appear to be less effective at reducing ghrelin levels than carbohydrates or proteins [13]. On the other hand, expanding the gastric volume with liquids such as water or saline/glucose does not modify circulating ghrelin levels [15]. In addition, several enteric neuropeptides secreted after nutrient intake, such as gastric somatostatin [16, 17] or insulin [18] also reduce ghrelin levels. We have previously shown that Ex-4, a potent GLP-1 receptor agonist, markedly reduces circulating ghrelin levels in fasting rats [19]. Other authors have shown that the vagal branch of the autonomic nervous system is a key factor in the regulation of ghrelin secretion in vitro [20] and in vivo [21–23]. All of these data together reveal that the mixture of signals activated during food intake, including sensory perception, differential nutrient composition, enteric postprandial secretion of neuropeptides, and metabolic signals, such as insulin and leptin [24], are implicated in reducing post-meal ghrelin levels.

With regard to the mechanisms mediating the increase in circulating ghrelin that anticipate hunger and the initiation of meals or its elevation with starvation, several studies have shown that β-adrenergic receptors are likely involved in ghrelin secretion *i*n vitro and in vivo, supporting the role of the sympathetic nervous system in controlling ghrelin secretion. Thus, norepinephrine increases ghrelin levels in primary cultures of stomach cells, whose effects are attenuated by β blockers [18, 25, 26]; while tyramine [27] stimulates the secretion of ghrelin in a specific tumor cell line (ghrelinoma) in vitro. In vivo, electrical stimulation of sympathetic nerves or their chemical activation with tyramine increases ghrelin levels in anesthetized Wistar rats [28]. Chronic administration of b-blockers (atenolol) reduces baseline ghrelin levels in 3-week-old mice [29] and fasting mice [28], and on the other hand deletion of B1 adrenergic receptors in ghrelin-producing cells reduced ghrelin levels [29] without affecting food intake or body weight.

In the present work we address the study of the pharmacological modulation of the β-adrenergic receptors, as effectors of the sympathetic branch of the ANS in the regulation of ghrelin levels in rats in vivo. We tested our hypothesis in rats that eat ad libitum and fasting, by different routes of administration (ip., iv., oral), in rats that were anesthetized and in freely-moving conditions. We studied ghrelin responses as a function of time and dose, and in relation to variations in glucose as a key metabolite regulated by b-adrenergic activation previously related to ghrelin regulation [30], and which serves as a positive control.

## Materials and methods

### Animals

Adult male Sprague-Dawley rats (250–325 g) from the colony of the University of Santiago de Compostela, were kept with free access to filtered water and standard food (A04; Panlab, Barcelona, Spain) with a 12-h light-dark cycle (lights on from 9:00–21:00) and controlled room temperature (20–21 °C), in the facilities of the bioexperimentation service of the University of Vigo. All experimental procedures were carried out in accordance with European Union regulations on the protection of animals used for experimental purposes (Council Directive EEC 86/609) and approved by the competent regional administration (CEIC, Xunta de Galicia, Spain, reference number: ES360570215601/17/INV. MED.02.OUTROS04/LCGM/02). Accordingly, the number of animals was reduced to 5–7 in each group, unless specifically stated otherwise.

**Fig. 1:**
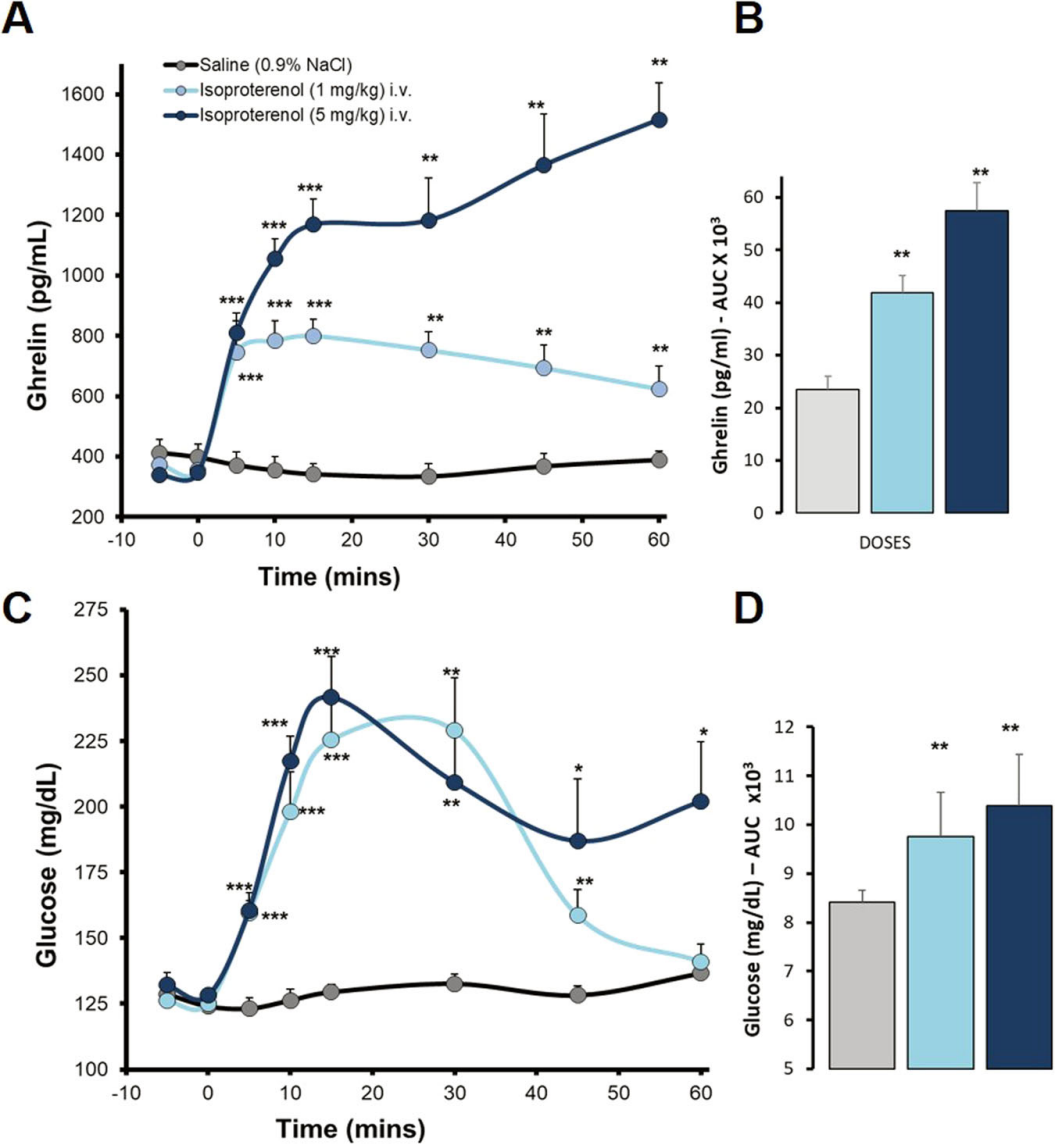
Dose-Response of intravenous administration of Isoproterenol on ghrelin and glucose levels in anaesthetized *ad libitum* fed rats. Intravenous administration. Data are means ± SEM, by Kruskal-Wallis test for multiple comparison, *P < 0.05, **P < 0.01, ***P < 0.001, treatment vs. saline. Groups: Saline (*n* = 7), Isoproterenol 1 mg/kg (*n* = 7), Isoproterenol 5 mg/kg (*n* = 7). **A**. Ghrelin levels (pg/mL) dose-response to isoproterenol. **B**. Area under the curve (AUC, pg/mlx1000) of ghrelin levels. **C**. Glucose levels (mg/dL) dose response to isoproterenol. **D**. Area under the curve (AUC, mg/dLx1000) of glucose levels

**Fig. 2:**
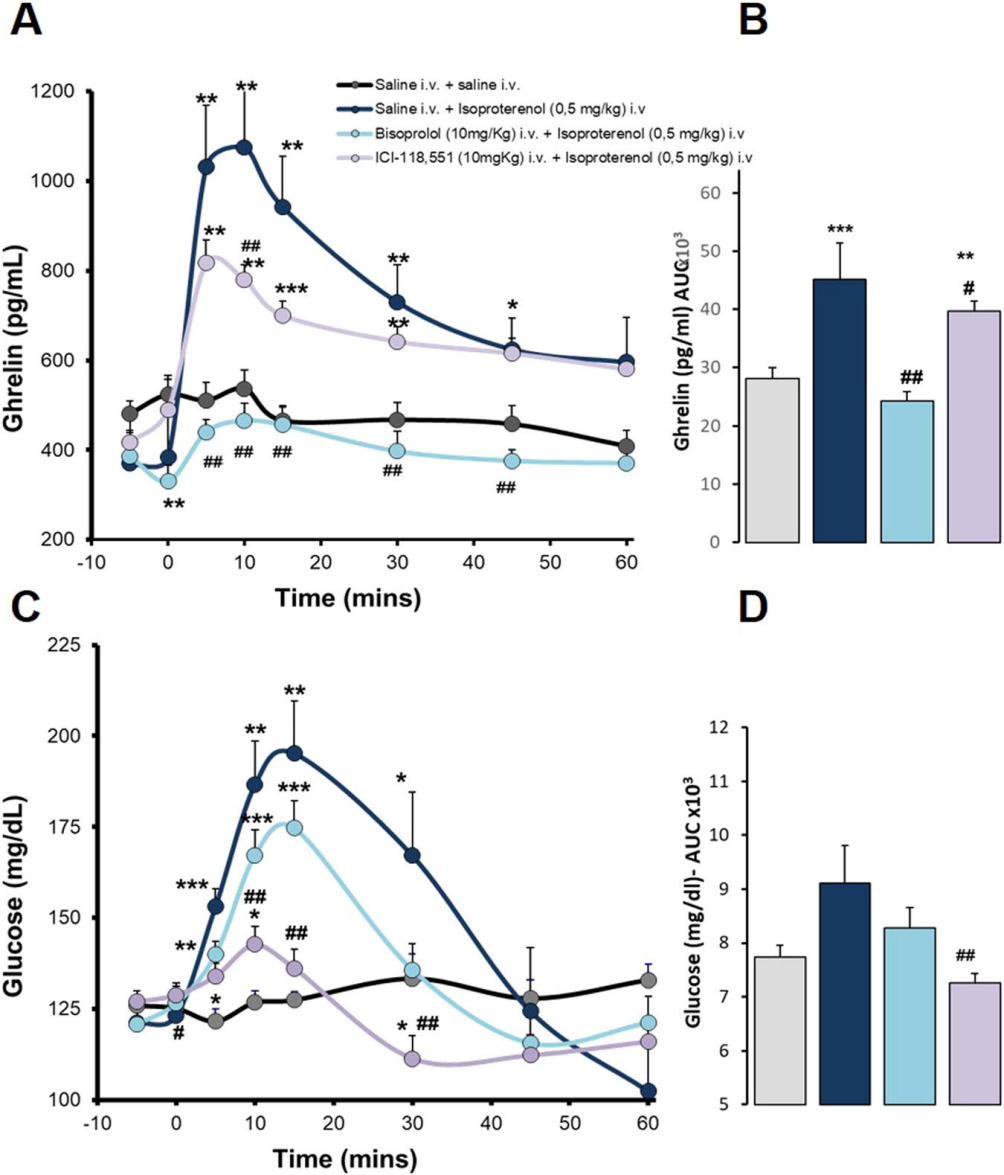
Effects of B-blockers in ghrelin and glucose responses to isoproterenol in anaesthetized *ad libitum* fed rats. Intravenous administration. Data are means ± SEM, by Kruskal-Wallis test for multiple comparison *P < 0.05, **P < 0.01, ***P < 0.001, treatment vs. Saline; #*P* < 0.05, ##*P* < 0.01 vs. Isoproterenol (ISO). Groups: Saline (*n* = 5), Isoproterenol 0,5 mg/kg (*n* = 5), Bisoprolol (10 mg/kg) + Isoproterenol 0.5 mg/kg (*n* = 7), ICI-118551 (10 mg/kg) +Isoproterenol 0.5 mg/kg (*n* = 7). **A**. Ghrelin levels (pg/mL) profile and effects of b-blockers in responses to isoproterenol. **B**. Area under the curve (AUC, pg/mlx1000) of ghrelin levels. **C**. Glucose levels (mg/dL) profile and effects of b-blockers in responses to isoproterenol. **D**. Area under the curve (AUC, mg/dLx1000) of glucose levels

**Fig. 3:**
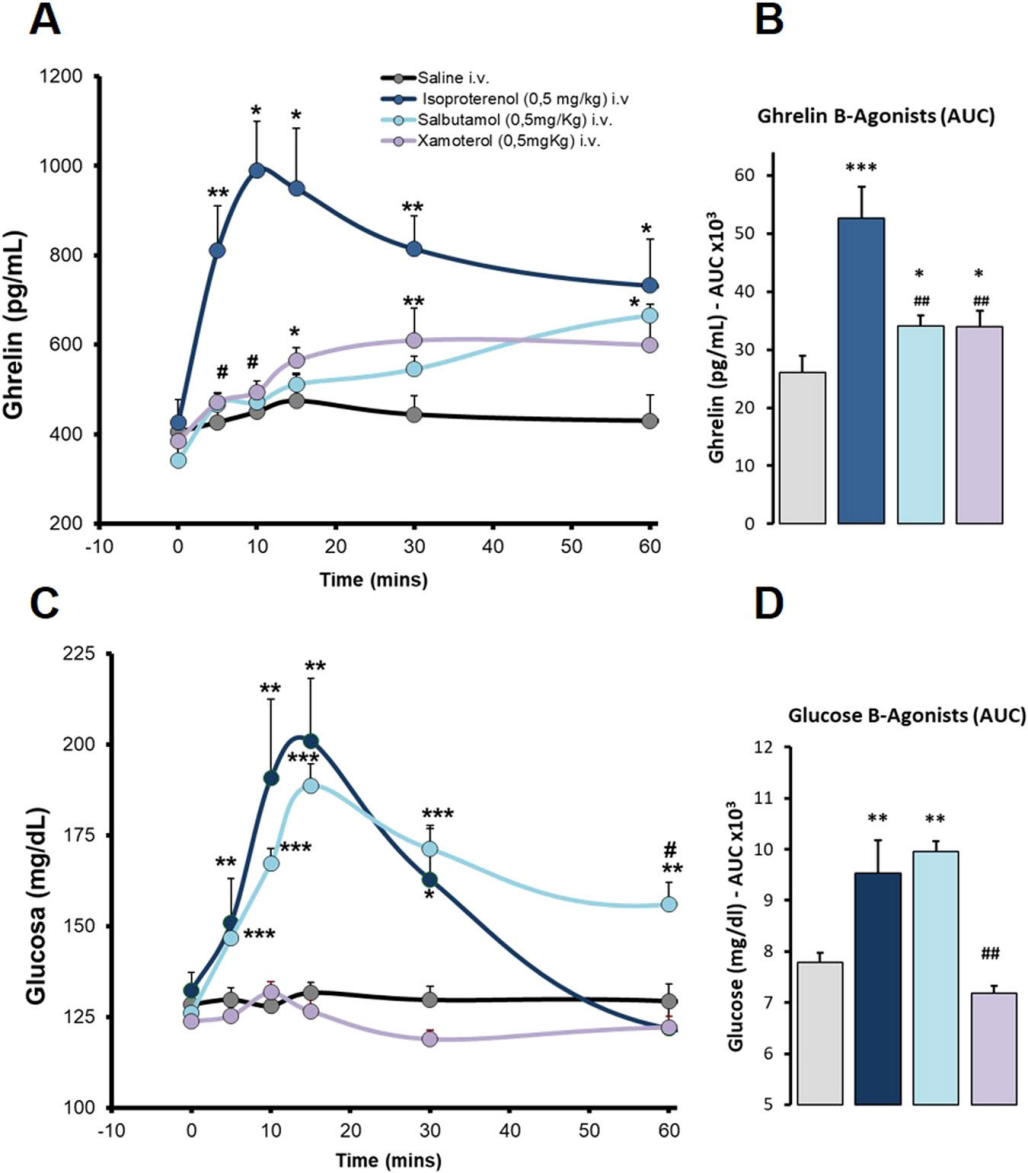
Effects of B-adrenergic agonists on ghrelin and glucose responses in anaesthetized *ad libitum* fed rats. Intravenous administration. Data are means ± SEM, by Kruskal-Wallis test for multiple comparison *P < 0.05, **P < 0.01, ***P < 0.001, treatment vs. Saline; #*P* < 0.05, ##*P* < 0.01 vs. Isoproterenol (ISO). Groups: Saline (*n* = 5), Isoproterenol 0.5 mg/kg (*n* = 5), Salbutamol 0.5 mg/kg (*n* = 7), Xamoterol 0.5 mg/kg (*n* = 7). **A**. Ghrelin levels (pg/mL) profile in response to B-adrenergic agonists. **B**. Area under the curve (AUC, pg/mlx1000) of ghrelin levels. **C**. Glucose levels (mg/dL) profile in responses B-adrenergic agonists. **D**. Area under the curve (AUC, mg/dLx1000) of glucose levels

A group of rats were fasted for 24 h before testing drugs to naturally increase circulating ghrelin levels, depending on the condition required for the same experiments. The rats were anesthetized with sodium pentobarbital (50 mg/kg CO3HNa2, Sigma-Aldrich, ip.) to access repeated blood samples through the right jugular vein, by inserting a silicone tip (inner diameter, 0.635 mm and outer diameter, 1.194 mm; Degania Silicone Ltd., Israel). Blood samples (300 μL) were taken at different times (baseline, 5, 10, 15, 30, 45, and 60 min) after administration of the adrenergic or vehicle modulators (0.9% NaCl, saline). Results are shown in Figs. 1–3. In some experiments, blood samples were taken from the trunk after sacrifice. Blood samples were collected on ice in pre-cooled tubes and immediately centrifuged (3500 rpm for 5 min at 4 °C), and serum was stored at −40 °C prior to processing and measurements.

### Intraperitoneal administration

**Fig. 4:**
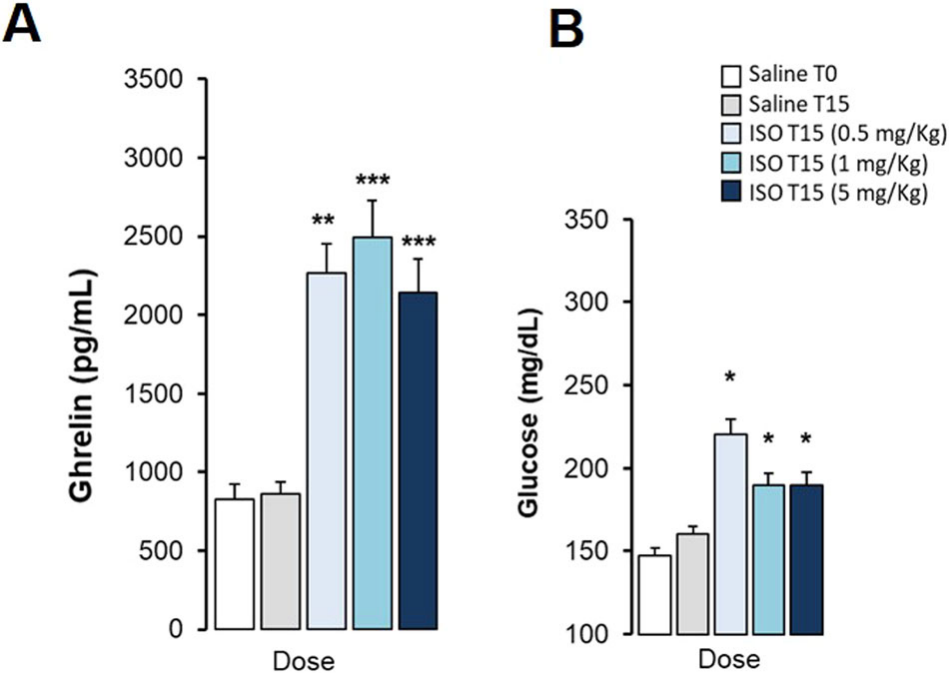
Dose-Response of intraperitoneal administration of Isoproterenol on ghrelin and glucose levels in freely-moving rats eating *ad libitum* up to the experiment. Intraperitoneal administration. Data are means ± SEM, by Kruskal-Wallis test for multiple comparison, * <0.05, **P < 0.01, ***P < 0.001, treatment vs. Saline T15. Groups: Saline T0 (*n* = 5), Saline T15 (*n* = 5), Isoproterenol 0.5 mg/kg (*n* = 5), Isoproterenol 1 mg/kg (*n* = 5), Isoproterenol 5 mg/kg (*n* = 5). **A**. Ghrelin levels (pg/mL) dose-response to isoproterenol. **B**. Glucose levels (mg/dL) dose-response to isoproterenol

**Fig. 5:**
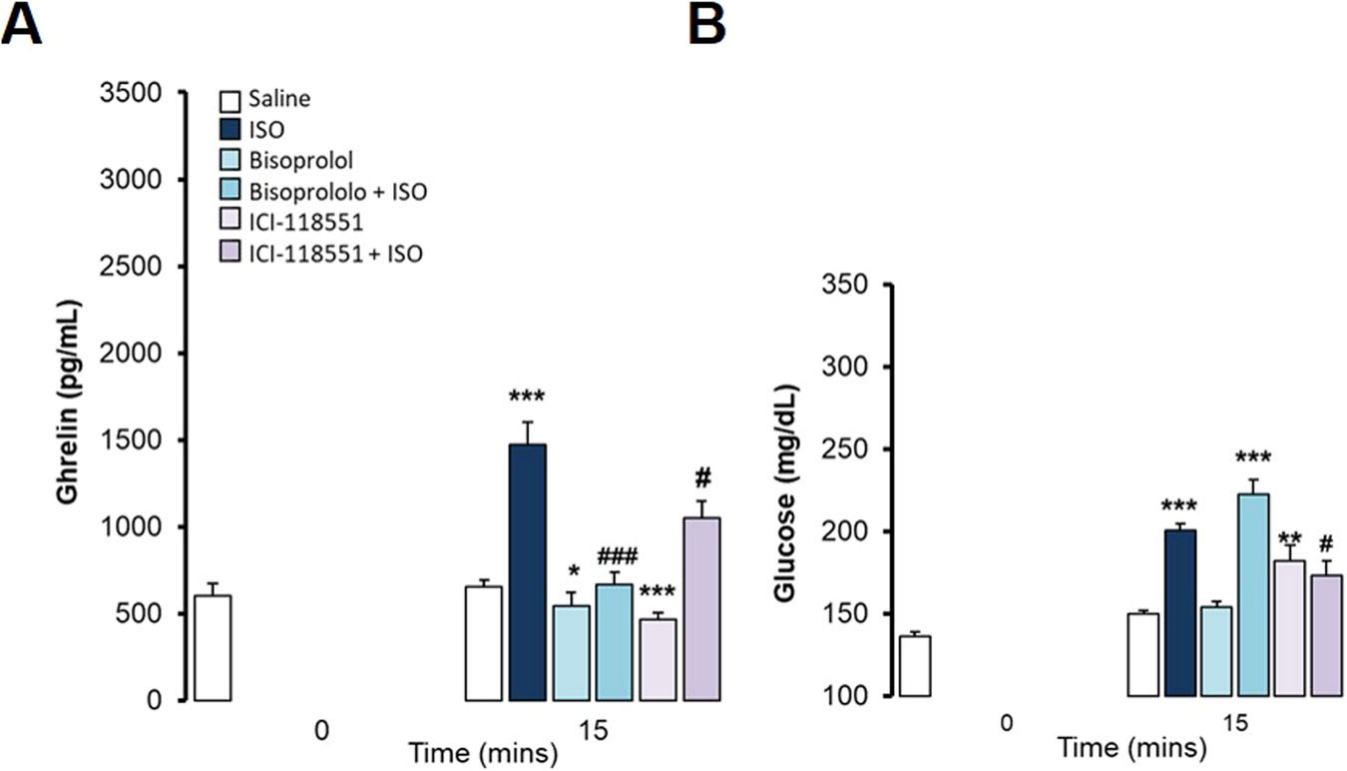
Effects of B-blockers in ghrelin and glucose responses to isoproterenol in freely-moving rats eating *ad libitum* up to the experiment. Intraperitoneal administration. Data are means ± SEM, by Kruskal-Wallis test for multiple comparison *P < 0.05, **P < 0.01, ***P < 0.001, treatment vs. Saline; #*P* < 0.05, ###*P* < 0.001 vs. Isoproterenol (ISO). Groups: Saline (*n* = 5), Isoproterenol 0.5 mg/kg (*n* = 5), Bisoprolol 10 mg/kg (*n* = 5), Bisoprolol (10 mg/kg) + Isoproterenol 0.5 mg/kg (*n* = 5), ICI-11855110 mg/kg (*n* = 5), ICI-118551 (10 mg/kg) + Isoproterenol 0.5 mg/kg (*n* = 7). **A**. Ghrelin levels (pg/mL) b-blockers effect in responses to isoproterenol. **B**. Glucose levels (mg/dL) effects of b-blockers in responses to isoproterenol

**Fig. 6:**
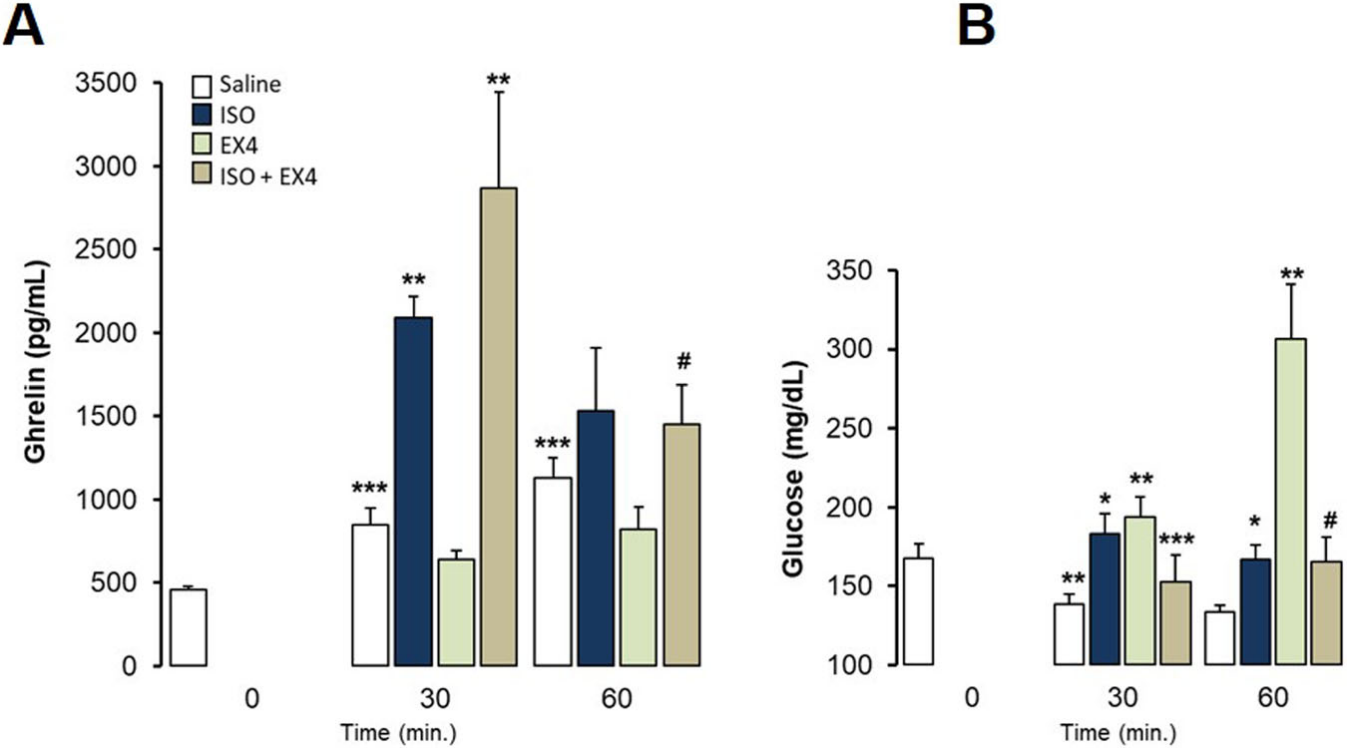
Interaction of Exendin-4 and Isoproterenol on ghrelin and glucose responses in freely-moving rats eating *ad libitum* up to the experiment. Intraperitoneal administration. Data are means ± SEM, by Kruskal-Wallis test for multiple comparison *P < 0.05, **P < 0.01, ***P < 0.001, treatment vs. Saline; #*P* < 0.05, ###*P* < 0.001 vs. Isoproterenol (ISO). Groups: Saline (*n* = 5), Isoproterenol 0.5 mg/kg (*n* = 5), Ex-4 5 μg/kg (*n* = 5), Isoproterenol 0.5 mg/kg + Ex-4 5 μg/kg (*n* = 5). **A**. Ghrelin levels (pg/mL) at 30 and 60 min after administration of drugs. **B**. Glucose levels (mg/dL) at 30 and 60 min after administration of drugs

**Fig. 7:**
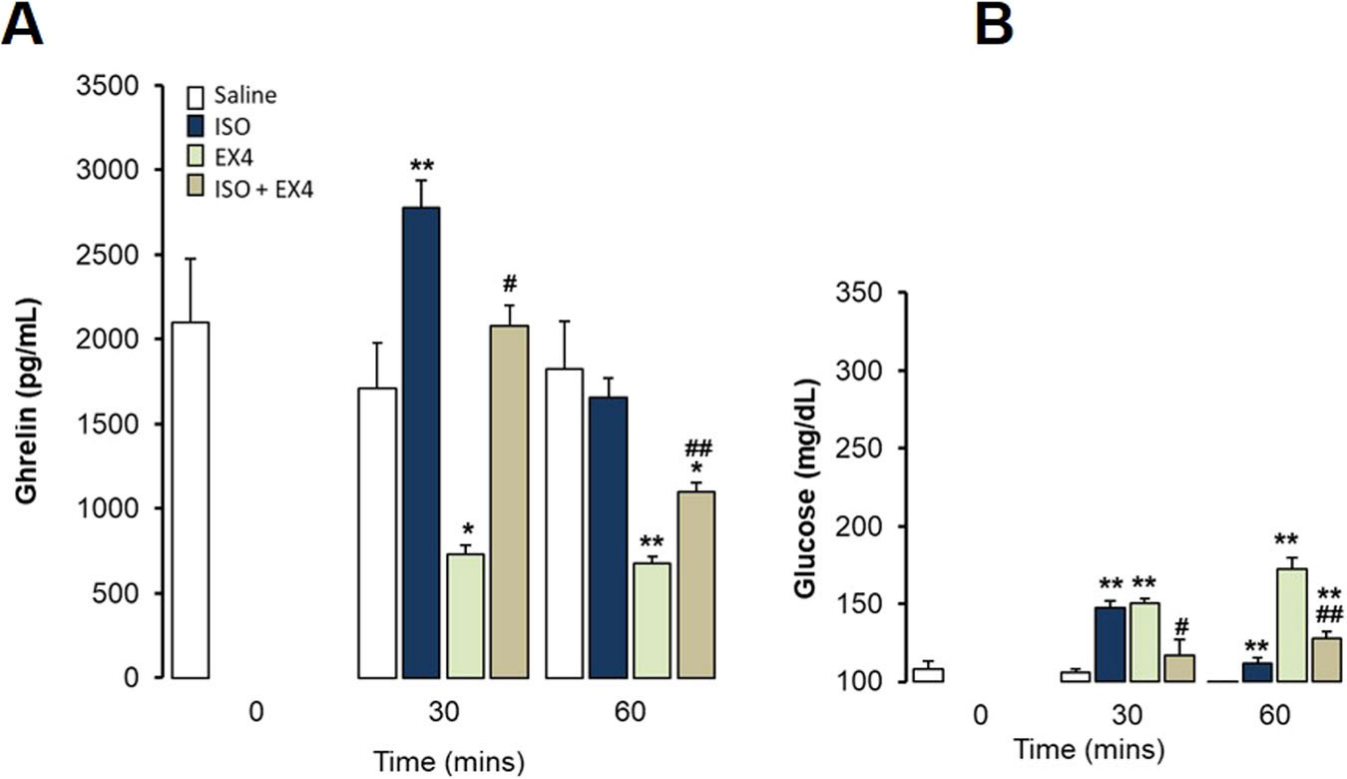
Interaction of Exendin-4 and Isoproterenol on ghrelin and glucose responses in freely-moving rats fasting 24 h before the experiment. Intraperitoneal administration. Data are means ± SEM, by Kruskal-Wallis test for multiple comparison *P < 0.05, **P < 0.01 treatment vs. Saline; #*P* < 0.05, ##*P* < 0.01 vs. Isoproterenol (ISO). Groups: Saline (*n* = 5), Isoproterenol 0.5 mg/kg (*n* = 5), Ex-4 5 μg/kg (*n* = 5), Isoproterenol 0.5 mg/kg + Ex-4 5 μg/kg (*n* = 5). **A**. Ghrelin levels (pg/mL) at 30 and 60 min after administration of drugs. **B**. Glucose levels (mg/dL) at 30 and 60 min after administration of drug

The rats were kept in current housing conditions described above before fasting, and the animals were handled daily to avoid stress. On the day of the experiment, the rats were administered intraperitoneally the test drug dissolved in sterile 0.9% saline solution or only the vehicle (saline), and returned to their cages. Blood samples from the trunk were collected after decapitation in ice-cooled EDTA tubes. The blood was centrifuged and the serum obtained was frozen and stored at −40 °C until it was analyzed. The results are shown in Figs. 4–7.

### Oral administration and effect in food intake

**Fig. 8:**
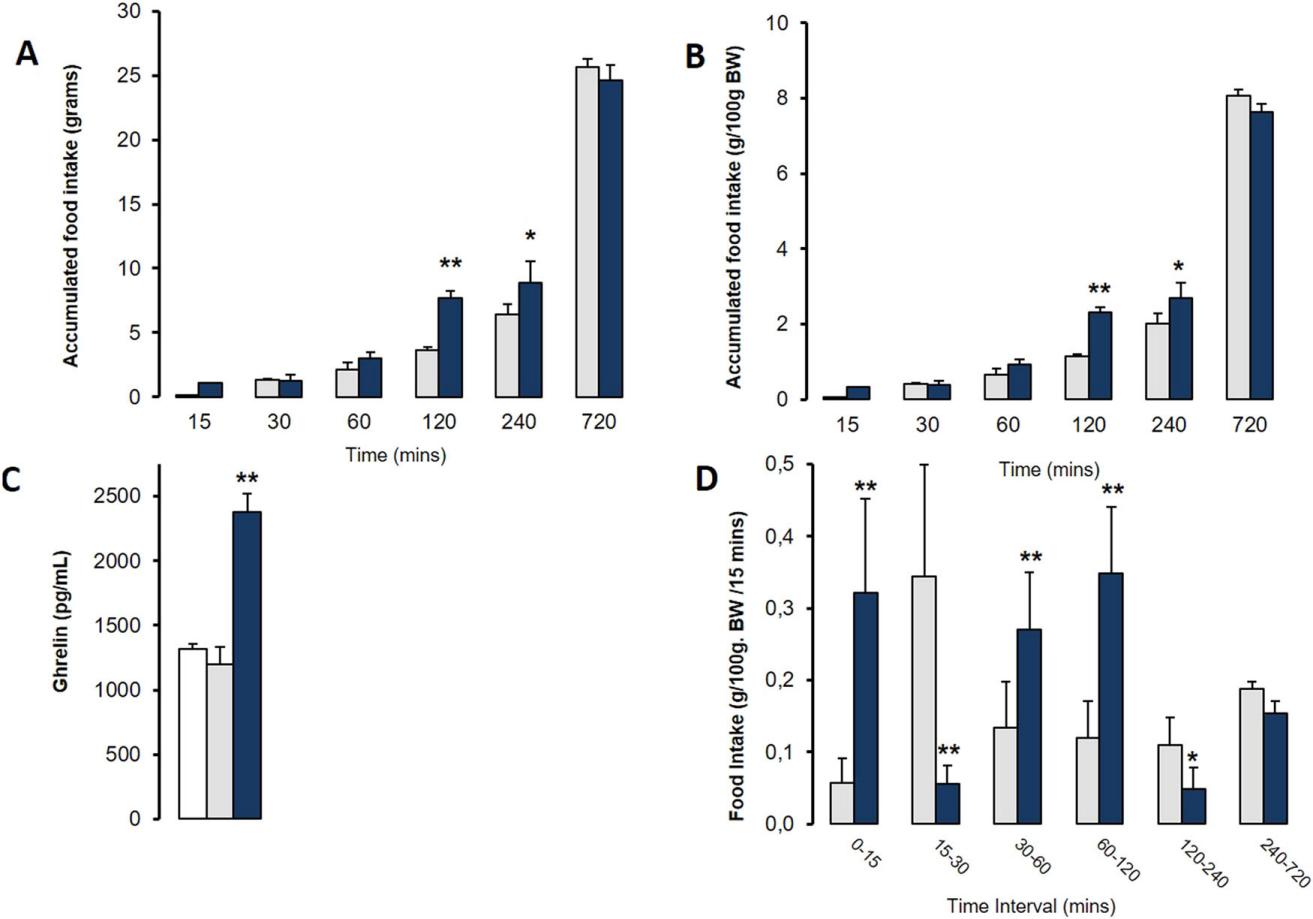
Effects of Isoproterenol (1 mg/mL) orally in drinking water available 60 min before time 0, on food intake and ghrelin levels in rats eating *ad libitum*. Data are means ± SEM, by Kruskal-Wallis test for multiple comparison *P < 0.05, **P < 0.01 treatment vs. Saline. **A**. Accumulated amount of food intake (*n* = 7). **B**: Accumulated amount of food intake per 100 gr of body weight (BW) of each rat (*n* = 7). **C**: Ghrelin levels at beginning of the experiment (*n* = 3). **D**: Food intake per 100 g of body weight (BW) per interval. Data are normalized to 15 min period for relative comparison of different length intervals (*n* = 7)

Male Sprague-Dawley rats (weight range: 275–325 g) were individually housed in metabolic cages (Tecniplast 3700 M071) for three days prior to the experiment to acclimatize and minimize environmental stress. On the day of the experiment, a bottle containing isoproterenol (1 mg/mL) solved in filtered water was added to the cage for ad libitum intake during 60 min and then removed right on the time to allow free access to the food when the lights were turned off. A quarter (n = 3) of the animals in each group were sacrificed to measure baseline ghrelin levels. Food intake was measured by the difference method weighing the food container at different times: 15, 30, 60, 120, 240, and 720 min after starting the experiments. The results are shown in graphs of Fig. 8 as measured and pondered by 100 g of body weight and time interval.

### Ghrelin and glucose measurements

Total ghrelin was measured using a specific radioimmunoassay (RIA) kit following the manufacturer’s instructions (RK-031-31, Phoenix Pharmaceuticals, USA). The intra-assay coefficients of variation (CV) were 2.5% (8 pg/tube), 2.0% (32 pg/tube) and 4.5% (128 pg/tube), while the inter-assay CVs were 7.2, 4.3 and 6.2%, respectively. Plasma glucose levels were determined by a spectrophotometric assay based on the glucose oxidase method with a commercial kit and following the manufacturer’s specifications (Glucose RTU, ref. 61269, bioMerieux, Fr). The coefficient of variation of all measurements was less than 2% over the range of concentrations of the assay (10–500 mg/dL).

### Drugs & substances

Isoproterenol hydrochloride (1-(3′,4′-dihydroxyphenyl)-2-isopropylaminoethanol hydrochloride. I-5627, Sigma-Aldrich, Spain), is a potent universal agonist activator of all β-adrenergic receptors. Bisoprolol (1-{4-{[2-(1-methylethoxy)ethoxy]methyl}phenoxy}-3-[(1-methylethyl)amino]-2-propanol, B2185, Sigma-Aldrich), is a long-acting selective β1-adrenergic receptor antagonist. ICI118551 hydrochloride, it is an highly selective antagonist of the β2-adrenergic receptors ((2R,3R)-rel-3-isopropylamino-1-(7-methyllindane-4-yloxy)-butan-2-ol hydrochloride; I127, Sigma-Aldrich, Spain). Xamoterol hemifumarate salt (N-[2-[[2-hydroxy-3-(4-hydroxyphenoxy)propyl] amino]ethyl]morpholine-4-carboxamide. X-3253, Sigma-Aldrich, Spain), is a specific partial agonist of β1 adrenergic receptors, with no agonist action on β2 adrenergic receptors. Salbutamol (α-[(tert-butylamino)methyl]-4-hydroxy-m-xylene-α,α‘-diol. S-8260, Sigma-Aldrich Spain), is a highly specific, short-acting β2 adrenergic receptor agonist. Exendin-4 (Ex4. E7144. Sigma-Aldrich (Spain) is a natural agonist of GLP-1 receptor agonists (Glucagon-Like Peptide-1) and a potent ghrelin inhibitor as previously reported [19].

### Statistical analysis and result representation

Data are represented as the mean ± standard error of the mean (SEM) in all charts, unless specifically stated otherwise. We compared the treatment vs. control groups (Vehicle, 0.09% NaCl saline) using the non-parametric Mann-Whitney test for independent samples. When more than 2 samples were compared, they were compared using the non-parametric analysis of variance of the Kruskal-Wallis test for multiple comparison. The AUC of the time-curve individual response was calculated using the trapezoidal rule. Statistical differences were conventionally accepted as significant when p < 0.05.

## Results

Intravenous (IV) administration of isoproterenol (ISO) to anesthetized *ad libitum* fed rats produced a marked dose-dependent increase in ghrelin levels (1 and 5 mg/kg) at all times studied after stimuli (5–60 min. Figure 1A). The area under the curve of the responses also reflected an increase in total ghrelin secretion, double and triple the baseline levels of control rats (saline) with the respective doses. Isoproterenol administration also increased glucose levels at all time points, but showed similar profiles at 1 and 5 mg/kg, indicating that those doses are sufficient to elicit maximal glucose responses, although they last longer at the highest dose (5 mg/kg). Figure 1B). This result indicates that glucose increases, per se, are not sufficient to block the increase in ghrelin levels, as previously suggested. Elevations in ghrelin levels were consistently elicited in both *ad libitum* and fasting animals.

We wanted to delve deeper into the β-adrenergic modulation of ghrelin secretion, so we challenged ghrelin with ISO to study the effects of specific β blockers. Bisoprolol, a specific β1-adrenergic blocker, prevented ISO-induced ghrelin elevations (Fig. 2A), at peak and at all time points studied in anesthetized *ad libitum* fed rats. On the other hand, iv. administration of ICI-188,551, a highly selective β2-adrenergic blocker, also partially reduced ISO-induced ghrelin elevations (Fig. 2A) at peak (T10) and throughout the time-response profile (M-W, p < 0.05). Those responses are also observed when represented by the AUC of ghrelin profiles. Bisoprolol completely blocked the effect of ISO to show no difference to controls treated with saline, while ICI-188,551 did so partially. In addition, as confirmation of the activity of the β-blocker, ICI-188,551 completely blunted ISO-induced glucose elevations, but bisoprolol only partially (Fig. 2B). In this experiment we used the lowest dose of ISO (0.5 mg/kg) which has been shown to be efficient enough to produce a high increase in ghrelin reaching a peak at 10 min, and more likely to be suppressed by the doses of β blockers used.

On the other hand, the administration of specific β-adrenergic agonists was not as conclusive. Salbutamol, a selective β2-adrenergic agonist, and xamoterol, a specific β1-adrenergic agonist, increased ghrelin levels in anesthetized ad libitum fed rats, although with less potency than ISO (Fig. 3C). Salbutamol significantly increases ghrelin levels compared to saline control at 5 and 15 min, and xamoterol at 10 and 60 min. Those elevations produced a slight increase in total ghrelin secretion, as shown by the AUC. ISO and salbutamol also greatly increased glucose levels, but xamoterol did not. Figure 3A, B respectively.

Since anesthesia could deeply change hormone regulation, we also tested ghrelin responses at 15 min (T15) to intraperitoneal (ip) administered ISO at different doses (0.5, 1, and 5 mg/kg) to conscious *ad libitum* fed rats. As shown in Fig. 4A, all three doses caused a marked elevation in ghrelin levels. Under these conditions, ISO also markedly increased circulating glucose levels (Fig. 4B). Again, in conscious rats, the effects of the β-blocker were similar to those shown in anesthetized animals, and BIS completely prevented ISO-induced ghrelin elevations and ICI was partially effective (Fig. 5A). In addition, BIS was ineffective in eliciting any changes in glucose per se neither in responses to ISO, but ICI slightly elevated glucose levels and partially blocked elevations in response to ISO (Fig. 5B).

We have previously reported that ghrelin levels can be very effectively reduced by administering Ex4 to fasting and *ad libitum* fed rats [18]. In the present work, ISO appeared to potently elicit ghrelin secretion (Fig. 6A). The ip. administration of Ex4 (5 μg/kg) was completely ineffective in preventing ISO-elicited increase in ghrelin levels in conscious *ad libitum* fed rats, even though Ex4 markedly raises glucose levels at 30 and 60 min (Fig. 6B), as we previously reported as well. ISO also increases glucose levels as has been shown in anesthetized animals, but interestingly, it antagonizes the effect of Ex4 on glucose control (Fig. 6B).

In addition, fasting itself increases basal ghrelin levels as expected, and Ex-4 ip. administration completely blocked this increase (Fig. 7A). In conscious fasting rats, ISO was able to further increase the already elevated ghrelin levels and completely avoid the inhibitory effect of Ex-4, which is a much highlighted effect. Again, in consciously fasting rats, ISO increases glucose levels on its own and also in the presence of Ex4, although both together have smaller effects than separately (Fig. 7B). Thus, ISO was able to increase ghrelin levels in both conscious fasting rats (Fig. 7A) and ad *libitum* fed rats (Fig. 6A), even in the presence of a potent inhibitor of ghrelin secretion such as Ex4, and despite changes in glucose levels (Fig. 7B).

A specific experiment also addressed whether ISO-induced ghrelin elevations would have any effect on food intake in *ad libitum fed* rats (Fig. 8). Food intake per 100 g of rat body weight (100 g/BW) showed that animals having ISO orally in drinking water have significantly higher intake in the intervals 0–15, 30–60, 60–120, but lower between 15–30, and slightly reduced in 120–240 (Fig. 8A). Rats given ISO showed higher levels of ghrelin when allowed access to food (Fig. 8C). This resulted in an increase in cumulative food intake and food intake by 100 g/BW at 120 and 240 min (Fig. 8B, D). Thus the increase in food intake is consistent with the increased ghrelin levels in the ISO group at the beginning of the experiment (Fig. 8C).

## Discussion

Ghrelin levels rose during fasting and dropped a few minutes after free food intake. Thus, variations in ghrelin levels appear to be related to periods of food intake [14]. The main source of circulating ghrelin is the fundus of the stomach, and variations in circulating ghrelin levels are closely associated with principal meals in animals and humans [4, 5, 31]. In fact, ghrelin has been attributed as the “hunger hormone” [9, 31]. However, the mechanisms underlying ghrelin variations are not fully understood. As circulating ghrelin levels depend on the gastric source, factors that regulate gastric activity must influence ghrelin production and secretion. Several different factors and hormones have been identified to reduce circulating ghrelin levels and inhibit its production and secretion from the gastric fundus. Despite previous research efforts in this field, key regulatory events that promote elevated circulating ghrelin levels before meals are still not fully understood. We have specifically studied and here reported the very important role of the beta-adrenergetic sympathetic pathway in explaining ghrelin elevations in rats in vivo. Beta-adrenergic activation is shown here as a very consistent stimulator of ghrelin elevations under all experimental conditions, regardless of feeding regimen (ad libitum/fasting), route of administration (ip, iv, oral), and free-moving or anesthetized animals.

The autonomic nervous system (ANS), as a major player in gastric regulation, has been shown to play a key role in ghrelin’s physiological responses to food intake, stress, and metabolites. The involvement of the ANS in ghrelin control was first described in sheep, in which ghrelin levels were shown to be higher after administration of atropine, a cholinergic blocker, or especially after hexamethonium, a general vegetative preganglionic inhibitor [21]. However, these results appeared to be opposite in rats, where atropine does not increase, but decreases fasting ghrelin levels [29, 32]. Vagal activation has been linked to ghrelin variations in humans as well, as vagal stimulation increased the inhibitory effect of fat on ghrelin levels in humans [33]. Thus, atropine reduced fasting ghrelin levels in healthy humans, and pyridostigmine, an acetylcholinesterase inhibitor that increases endogenous cholinergic tone, did not modify [22] or increase [23] ghrelin levels. Moreover, antagonism of vagal activity by sympathetic nerves promotes ghrelin elevations in vivo [20]. Accordingly, supradiaphragmatic vagotomy prevents the fasting-related increase in plasma ghrelin, but does not modify the inhibitory response to nutrients, leading to the conclusion that inhibition and elevation of ghrelin are due to different mechanisms [32, 34].

In addition, the sympathetic branch of the ANS in relation to ghrelin secretion was also explored. In vivo activation of sympathetic nerves, as might happen in stress-induced responses [35], has been shown to increase circulating ghrelin levels [28]. In vitro studies showed that norepinephrine stimulates ghrelin secretion in primary cultures of stomach cells [14, 18, 28] and in ghrelinoma cell lines from mice [25, 27], suggesting that those effects may be mediated by β-adrenergic receptors. In vivo studies have also shown that chronic administration of the β1 blocker atenolol reduces the elevation of ghrelin levels in mice, but does not affect food intake [29]. Our results are in agreement with the previous ones and here we describe a consistent and dose-dependent acute increase in ghrelin induced by Isoproterenol, a potent universal b-adrenergic agonist capable of binding with similar affinity to β1, β2 or β3 adrenergic receptors. These increases occur when ISO was administered intravenously to anesthetized animals (Fig. 1A, B) and by intraperitoneal injection to conscious rats (Fig. 4A). The ISO-induced increase in ghrelin was completely suppressed by bisoprolol, a known selective β1 antagonist. Interestingly, ICI-118551, a selective β2-adrenergic receptor blocker, was also able to significantly reduce ghrelin responses to ISO (Fig. 2) at peak (10 min) and area under the curve, revealing that β2-adrenergic receptors may be secondarily involved in ghrelin increases. Both adrenergic agonists, β1 xamoterol and β2 salbutamol, induced a similar modest but significant increase in ghrelin levels, albeit of a much smaller amplitude than that induced by ISO (Fig. 3A, B), suggesting that activation of both adrenergic receptors may be necessary to achieve a complete response on ghrelin.

Another set of possible regulatory factors of ghrelin secretion are metabolic signals, including energy substrates whose levels are modified after food intake. In fact, glucose levels have previously been implicated in ghrelin inhibition [30]. Thus, we measure glucose levels for a dual purpose: as the main energy substrate previously related to variations in ghrelin levels, but also as a marker of the activity of the different adrenergic modulators. In addition, increased glycaemia is a characteristic response of β2-adrenergic receptors that helps to distinguish the selectivity of β-blockers in vivo [36, 37]. Therefore, changes in glucose levels may interfere with the interpretation of ghrelin responses to β-adrenergic modulators. Unsurprisingly, ISO greatly increased glucose levels in a dose-dependent manner (Figs. 1C, D, and 4B). The data shown in Fig. 2 confirm that ICI-118551 almost completely blocked ISO-induced glucose elevations, whereas bisoprolol did so very partially, confirming that bisoprolol is a highly selective β1-blocker although it may retain some β2-blocking activity. In the case of β-agonists, their responses were completely independent of glucose variations, as the β2-agonist salbutamol induced a marked increase in glucose levels similar to ISO, as expected, while xamoterol, as β1-agonists, did not change the glucose levels respect to the control group (Fig. 3C, D). Since then, we have been able to show that variations in circulating glucose levels depend fundamentally on β2-adrenergic activation. It should be mentioned that the agonist/antagonist capacity of different molecules with respect to beta-adrenergic receptors is not as completely specific, and they may retain some slight activity in the other receptors. In addition, it has been widely observed that an agonist activity in a specific type of receptor is usually accompanied by partial antagonism to each other, especially as has been studied in adrenergic receptors [38, 39]. In fact, previous studies on ghrelin responses to β-blocker were conducted with atenolol, which despite being used as a selective β1-blocker retains some β2-blocker activity sufficient to increase glucose levels and insulin demand [40, 41]. Atenolol is less selective to β1-adrenergic receptor than the bisoprolol we used [38], and for that reason previous studies attributing the capacity to increase ghrelin circulating levels specifically to the β1-adrenoceptor should be carefully considered [40]. In addition, the offspring of mice with deletion of the adrenergic β1-receptor in ghrelin-secreting cells retain some ghrelin-increasing response to norepinephrine [29].

Several neuropeptides modified by meals could also activate the central pathways that control peripheral ghrelin secretion. We previously described that Ex4, a GLP-1 receptor agonist, potently decreases ghrelin secretion and production, supporting this as a complementary mechanism by which incretins and the GLP-1 receptor agonists (GLP-1RAs) participate in the control of food intake as a sign of satiety [19]. Here we show that β-adrenergic activation increased the circulating levels of ghrelin even in the presence of Ex4, reversing its potent inhibitory effect, regardless of the dietary pattern both in fasting (Fig. 7A) and in rats *ad libitum* fed (Fig. 6A). It is very remarkable that fasting is a potent stimulus to increase ghrelin levels, and even in that condition ISO additionally increased ghrelin levels avoiding the inhibitory effect of Ex-4 and independently of glucose variations (Figs. 6B and 7B).

Finally, changes in ghrelin circulating levels may or may not modify the food intake regime. In our latest experiment, we have shown that isoproterenol administered in “drinking water” *ad libitum* to rats markedly increases basal ghrelin levels and food intake in the following hours. We show that cumulative food intake and weighted food intake by 100 g/BW, but also the rate of intake (food intake by time interval), increases in rats within two hours of exposure to isoproterenol. These data suggest that β-adrenergic tone modulation is a very relevant signal for ghrelin induction and involved in the initiation of food intake in the short term, and that the activation of the sympathetic nervous system plays a key role in raising ghrelin levels before meals.

Our study may have some limitations. Specifically, responses in humans may be different from those shown here in experimental models, but consistent induction of ghrelin by ISO in all conditions, conscious and anesthetized animals, fasting and ad libitum feeding, by all routes of administration (iv., ip., oral), in a dose-dependent manner and avoiding potent inhibitory signals such as GLP-RAs, allows us to rely on the translation to human responses as currently shown with other studies on the regulation of ghrelin. In addition, there are known to be two different molecular forms of ghrelin: acyl-ghrelin (AG) and deacylated ghrelin (DAG). There is only one receptor identified to mediate ghrelin responses named GH-secretagogue receptor-1 (GHSR-1a). Thus, until recently, acyl-ghrelin was considered the biologically active molecule and deacylated ghrelin as a degradation metabolite generated by the GOAT (ghrelin O-acyl-transferase) enzyme that is present in gastric secretory ghrelin cells and in many other ghrelin target cells. However, recent studies have shown that DAG may have some biological effects acting on GHSR-1a as a partial agonist but as an antagonist in most cases by blocking some ghrelin responses, and finally acting through other unidentified receptors [42]. We have measured total ghrelin, therefore, we have not differentiated between the circulating levels of both molecular forms of ghrelin, whose levels can vary differentially depending on the regulation of GOAT: commonly increases in ghrelin are associated with reductions in DAG. Therefore, differences in circulating levels of ghrelin and DAG are more likely to be due to changes in GOAT regulation than in ghrelin secretion from gastric cells. Addressing the study of the differential secretion of ghrelin isoforms in vivo would force a whole series of new experiments, which would justify future studies. In summary, activation of the sympathetic branch of the autonomic nervous system through β-adrenergic receptors is a key mechanism necessary for elevation of ghrelin levels in fasting, but also in *ad libitum* feeding, and involves β1 and β2 adrenergic receptors. It is a very potent mechanism, independent of glycemic control and powerful enough to overcome the marked inhibitory effect of GLP-1RAs on ghrelin secretion. Our results emphasize that beta-adrenergic-induced increase in ghrelin may have a physiological role regardless of diet regimen and contributes to explaining how events that stimulate sympathetic activity physiologically, such as intense exercise [6], might be related to increased food intake and appetite. Moreover, the results shown here may be of potential interest in the clinic, for example in those cases with characteristic low ghrelin levels, such as in oncological cachexia [10, 11].

## Data Availability

Original datasets are available upon reasonable request to corresponding author.
